# Validation of an English version of the Child-OIDP index, an oral health-related quality of life measure for children

**DOI:** 10.1186/1477-7525-4-38

**Published:** 2006-07-01

**Authors:** Huda Yusuf, Sudaduang Gherunpong, Aubrey Sheiham, Georgios Tsakos

**Affiliations:** 1 Department of Epidemiology and Public Health, University College London, UK; 2 Department of Community Dentistry, Faculty of Dentistry, Chulalongkorn University. Bangkok, Thailand

## Abstract

**Background:**

To evaluate the psychometric properties of the Child-OIDP for use among children in the UK and report on the prevalence of oral impacts in a sample of schoolchildren in Westminster.

**Methods:**

Children aged 10–11 years in the final year of primary school (year 6) were selected from seven schools where annual screenings are carried out. A total of 228 children participated (99% response rate). A clinical examination was conducted followed by a questionnaire designed to measure oral health-related quality of life in children, namely the Child-OIDP. The psychometric properties of the Child-OIDP were evaluated in terms of face, content and concurrent validity in addition to internal and test-retest reliability.

**Results:**

The Child-OIDP revealed excellent validity and good reliability. Weighted Kappa was 0.82. Cronbach's alpha coefficient was 0.58. The index showed significant associations with perceived oral treatment needs and perceived satisfaction with mouth and oral health status (p < 0.001).

**Conclusion:**

This study has demonstrated that the Child-OIDP is a valid and reliable index to be used among 10–11 year old schoolchildren in the UK.

## Background

The concept of need is central to planning, provision and evaluation of health care services. Traditionally, need has been estimated by using professionally based measures, known as normative need. Although normative need is important, it mainly reflects the clinical aspects of illness. However, subjective measures of health are important too, because they provide insights into how people feel and how satisfied they are with their quality of life [1]. Health-related quality of life instruments should therefore be used in conjunction with clinical measures.

A child's oral health can impact on eating, smiling, speaking and socialising. Oral conditions, such as dental caries may result in pain, which in turn may lead to consequences on a child's daily life such as taking time off from school or difficulty eating. Facial appearance and its relation to body image, self-esteem and emotional well-being also play important roles in social interaction. Measuring oral impacts in children is particularly relevant, as it will aid researchers and policymakers in assessing need, prioritising care and evaluating treatment outcomes [2]. Furthermore, children do not live in isolation; children's oral conditions may affect their siblings and parents. Studies using the lifecourse approach have highlighted that dental conditions have wider repercussions not only for the present but also in adulthood [3]. These issues have stimulated current interest in paediatric research related to quality of life.

Although there has been an increase in the development and use of oral health related quality of life measures in the past two decades, most have been developed for use in adults. Very few have been developed specifically for or used in children [4-7]. This is because there are numerous methodological and conceptual problems when developing paediatric health-related quality of life measures. For example, children's understanding of illness and health is age dependent due to social, language, emotional, and cognitive development [8]. Children undergo changes in psychosocial awareness, physical changes in dental and facial features, as well as cognitive developments [5,8]. These occur at certain stages of life and hence measuring and comparing these changes at different ages may be difficult. This study has been carried out in an attempt to contribute to the development and application of valid and reliable oral health-related quality of life measures in children at a particular age.

The Child Oral Impacts on Daily Performances (Child-OIDP) was developed and tested among 11–12 year old Thai children [5] to assess the prevalence and severity of impacts and factors related to the impacts. This index can also be used to assess oral health needs in population surveys, thus making it useful for planning services. Its scoring system enables health planners to prioritise dental care according to the severity of impact scores of subjects. It has been found to be a valid and reliable index among children in Thailand [5] and in France [9]. Its use in different countries and age groups is advocated. In order to validly use the instrument in the UK, it is important to investigate its psychometric properties. The main objective of this study is to evaluate the psychometric properties of the Child-OIDP for use among children in UK. In addition, we will also report on the prevalence of oral impacts in a sample of 10–11 year old schoolchildren in Westminster, London.

## Methods

The study was carried out on an opportunity sample selected from children attending state schools in Westminster, London. This is an inner-city London area with a culturally diverse population. Seven state schools that were covered by the dental clinic, where the principal author (HY) worked, were selected. All seven primary schools were chosen for the study. The schools were targeted annually for dental screening and the data collection in each school was carried out at the end of the school screening. The sample included 10–11 year old children in the last year of primary school (year 6). All 232 boys and girls in year 6 were asked to participate.

Before the main study, a pilot study was carried out on children of the same age in a different area in London. It confirmed the feasibility of the methodology with only minor modifications of the wording of the questionnaire.

The study had two aspects: a clinical examination followed by the administration of a questionnaire. Clinical examinations were carried out by the principal investigator (HY) with the help of a dental nurse, who acted as a recorder for the examination and was further involved in the administration of the questionnaire. The administration of the questionnaire involved face-to-face interview with each child on an individual basis. We will not present the clinical results in this publication. The socio-demographic and oral health-related quality of life data were collected through interview-administered questionnaires. Re-examinations were carried out (one week later) on oral health-related quality of life (OHRQoL) data on 18 children, representing 8% of the sample.

The Child-OIDP questionnaire was the measure of oral-health related quality of life used in this study [5]. It is derived from the OIDP with wording modifications addressing children's capability in relation to their intellectual, cognitive and language development. It is based on a modified version of WHO's International Classification of Impairments, Disabilities and Handicaps [10,11]. The Child-OIDP assesses oral impacts on the following daily performances: eating, speaking, cleaning teeth, smiling, emotional stability, relaxing, doing schoolwork, and social contact. In the Thai version, pictures were used to portray the impacts for better understanding. These were not used in this study, as the pilot study confirmed that children were able to understand the relevance of the impacts without needing pictures. Participants were asked about the frequency and severity of each impact on Likert scales (0 to 3)*.

Every time a scale is used in a new context or with a different population group, it is necessary to test its psychometric properties [12]. The psychometric testing involved the assessment of internal and test-retest reliability in addition to face, content and concurrent validity. Face validity refers to whether the items appear to be measuring what they are supposed to measure. Content validity is similar to face validity, the main difference is that a panel of experts examines the instrument and determines the degree to which its items address the topics the instrument is supposed to measure. This means looking at reading level and vocabulary [12]. Since there is no gold standard OHRQoL index, criterion validity is not assessed. In such a case, it is recommended that the validation process should rely heavily on concurrent validity [13] which examines a logical hypothesis by testing the index against a proxy measure of a similar concept. To assess the concurrent validity of the Child-OIDP, a number of questions were asked on perceived oral health and satisfaction with mouth, and the perceived need for dental treatment.

Ethical approval was given by the Local Ethics Committee. Parents and head teachers were sent information sheets regarding the purpose of the study. Negative consent was sought from the parents, as this was common practice for school dental inspections. In addition, positive consent was sought from the children.

### Data analysis

Internal reliability was tested by using the standardised Cronbach alpha coefficient, as well as item-total and inter-item correlations. Test-retest reliability was tested by using the weighted kappa for categories of the Child-OIDP scores, as well as the intra-class correlation coefficient (ICC) using the two way random effects model for the Child-OIDP score.

Face and content validity were empirically tested before and after the pilot study. The frequency distribution for the Child-OIDP scores is skewed and hence non-parametric testing was applied. Consequently, testing for concurrent validity referred to the relationship between the Child-OIDP and the following variables:

Perceived satisfaction with mouth (re-categorised into three categories; "low", "moderate" and "high") using the Kruskal-Wallis test, perceived oral health and treatment need (re-categorised into binary variables) using the Mann-Whitney test.

The data were analysed using the SPSS statistical package. The cut-off level for statistical significance was set at 0.05.

## Results

232 children were invited to participate in the study and 228 agreed. Three children did not give consent and 1 child did not speak English. The response rate was 99% (228/231 children).

Overall, 40.4% of participants reported at least one oral impact affecting their daily performance in the past three months (Table 1). The most prevalent impact was difficulty eating (23.2% of children), followed by impacts on cleaning teeth (18%), emotional stability (11.8%) and smiling (9.6%). Doing schoolwork and social contact were the least prevalent impacts, occurring in 1.8% and 2.2% of children in the sample.

**Table 1:** Prevalence of oral impacts on daily performances (Child-OIDP)

Oral Impacts on Daily Performances	% of children (N = 228)
Eating	23.2%
Cleaning teeth	18.0%
Speaking	3.9%
Smiling	9.6%
Relaxing/sleeping/	7.0%
Emotional stability	11.8%
Doing Schoolwork	1.8%
Social contact	2.2%
**Any Impact**	40.4%

In terms of internal reliability, the inter-item correlation coefficients among the 8 items of Child-OIDP ranged from -0.04, which represented the relationship between sleeping and cleaning, to 0.54 (Table 2). The vast majority of the inter-item correlations were positive, but very few correlations were negative, but very close to zero. The corrected item-total correlation coefficients ranged from 0.12 (doing homework, social contact) to 0.49, which related to emotional stability (Table 3). The standardised Cronbach's alpha coefficient was 0.58. Furthermore, the alpha coefficient did not increase when any of the items were deleted.

**Table 2:** Reliability analysis: Inter-item correlation for the Child-OIDP

Performance Scores	Eating	Emotion	Cleaning	Schoolwork	Speaking	Social Contact	Smiling	Sleeping
Eating	1.00							
Emotion	0.41	1.00						
Cleaning	0.30	0.36	1.00					
Schoolwork	0.16	0.15	0.00	1.00				
Speaking	0.00	0.04	0.14	-0.01	1.00			
Social Contact	0.07	0.07	0.03	-0.01	0.26	1.00		
Smiling	0.16	0.35	0.25	-0.01	0.27	-0.03	1.00	
Sleeping	0.14	0.05	-0.04	0.12	0.54	0.19	0.10	1.00

**Table 3:** Reliability analysis: Corrected item-total correlations

**Items**	**Corrected item-total correlations**	**Alpha item if deleted**
Cleaning teeth	0.36	0.51
Social contact	0.13	0.58
Eating	0.38	0.51
Emotional stability	0.49	0.47
Doing schoolwork	0.12	0.58
Sleeping	0.20	0.56
Smiling	0.34	0.52
Speaking	0.29	0.55

Standardised item alpha = 0.58

Test-retest reliability is the degree of agreement between two measurements taken at two different points in time using the same scale and with the same respondents; this provides an estimation of the degree to which the results are reproducible [14]. In this study, the weighted kappa statistic was 0.82 and the ICC was 0.88.

Face and content validity of the Child-OIDP were established prior to the main study. During the pilot study, the Child-OIDP questions were administered to a sample of 20 children. In addition, the relevance and understanding of the questionnaire was verified through a discussion with the children and their teacher. As a result, very minor changes were introduced prior to the main study.

In relation to concurrent validity (Table 4), those with a higher Child-OIDP score were less likely to be satisfied with their mouth (p < 0.001). Similarly, those who perceived their oral health as fair or poor are more likely to have a higher Child-OIDP when compared to those that perceived their oral health as "good", "very good" or "excellent" (p = 0.01). Furthermore, children who perceived a need for dental treatment had much higher Child-OIDP scores than those who did not have perceived need (p < 0.001).

**Table 4:** Concurrent validity tests for the Child-OIDP: comparison of Child-OIDP scores between different categories of related outcome variables

**Variables **(Categories)	N.	Child-OIDP Quartiles	P-value
**Perceived Oral Health **^1^			
Fair or poor	91	(0, 1.4, 5.6)	0.01
Good, very good or excellent	137	(0, 0, 2.8)	
**Perceived satisfaction with mouth **^2^			
Dissatisfied	29	(0, 5.56, 11.81)	<0.001
Neither/nor	53	(0, 0, 4.17)	
Satisfied	146	(0, 0, 2.78)	
**Perceived Dental Treatment Need **^1^			
Not Present	150	(0, 0, 1.7)	<0.001
Present	78	(0, 1.4, 8.3)	

^1 ^Mann-Whitney, ^2^Kruskal-Wallis

## Discussion

This study showed that the Child-OIDP index has good reliability and excellent validity among a culturally diverse sample of 10–11 year-old children in Westminster, thus indicating its applicability for child populations of similar ages in UK.

Test-retest reliability was confirmed as both the weighted kappa (0.82) and the ICC (0.88) indicated very good reliability. In terms of internal reliability analysis, the majority of corrected item-total correlations were above the recommended level of 0.2 [15], with the exception of those for social contact and doing schoolwork. Also, nearly all inter-item correlations were positive and no correlation was high enough for any item to be redundant. Some of the inter-item correlations were negative, but very close to zero, most of which related to doing schoolwork and social contact. However, this should be expected, as the same two items ('social contact' and 'doing schoolwork') were also the least prevalent. This might be due to the fact that children in this particular age group do not attach much importance to those activities. An alternative explanation is that enjoying contact with people might be an inherently unstable construct to the children, which varies with time [16].

The Cronbach alpha coefficient was 0.58 and this value did not increase when any item was deleted. This value may be questioned as some authors have recommended a value of 0.70 [15]. But, when examining internal reliability coefficients, scepticism is advised regarding what they are supposed to demonstrate, with a close examination of the item and subject conditions [17]. The criterion for "adequate" internal reliability depends on the purpose of the measure. For purposes of group comparisons, reliability does not have to be as high as it would have to be to make individual comparisons [18]. Reliability of 0.5 or above is considered to be acceptable [19-21].

On an important methodological point, the over-reliance on the actual value of Cronbach alpha for the assessment of reliability of an index should be open to further debate. This value is dependent not only on the magnitude of the correlation among the items but also on the number of items in the scale [12]. A scale can be made to look more homogenous and obtain high value of average internal correlation (Cronbach alpha) simply be doubling the number of items or adding a similar set of items [18]. Lower values of Cronbach alpha can be expected from shorter scales [22]. The Child-OIDP, which has few items (less than 10), falls into this category. Thus, a questionnaire with fewer items will be less internally consistent as each item is less relevant to the others and will result in patient scores that fluctuate more, due to random responses, in comparison to a longer instrument where a few items can be closely related (e.g. eating, drinking and chewing) and thus obtain a high value of Cronbach alpha [23]. Hence, it is not sufficient to simply compare Cronbach alpha levels when looking for a reliable instrument, because the alpha level will be lower in instruments with fewer items [12,22,24]. Higher reliability coefficients cost more than lower ones since they require more items. This poses a trade off between a brief and internally consistent measure that could be taken into a consideration in order to improve the practicality of an index. This is especially so for indices designed to be used at a population level and to obtain good cooperation of subjects, children in particular. They may not cooperate well with a long index. It is important that OHRQoL measures should be as brief as possible and user friendly in order to reduce the time and cost burden of to researchers and children, yet capturing all the dimensions related to OHRQoL [25,26].

The psychometric properties of instruments are dependent on the linguistic and cultural context in which they are used, especially as health is dynamic and depends on the environment. The face and content validity of the Child-OIDP was established in the pilot study. Concurrent validity was tested demonstrating significant relationships between Child-OIDP scores and satisfaction with mouth, perceived oral health and perceived dental treatment need. These results emphasise that perceptions of oral health and satisfaction with the mouth are strongly associated with oral health-related quality of life; the better the perception, the lower the prevalence of oral impacts.

Overall, 40% of children had an oral health related impact on their daily performance. This is lower than in other studies of similar ages [5,9,27]. This could partly be explained by different disease levels, age groups, culture and location of the sample. The most prevalent impact was 'eating' which was consistent with findings on other populations using the OIDP and Child-OIDP [5,9,27].

The importance of oral health-related quality of life is particularly relevant for children. Their perceptions are important as a number of their social and psychological coping skills are still developing. Because of their stage of development, they may be more sensitive to a variety of impacts, such as appearance, on their health-related quality of life. These impacts will affect their current quality of life and psychological development and may ultimately result in influencing their social skills and education [3,7]. An understanding of OHRQoL can only be achieved by asking the child about the impact of dental conditions on their quality of life. Although this may be complicated in children due to developmental issues [8], the use of paediatric OHRQoL measures should be encouraged in order to gain insights into the full impacts of dental illness and health.

Children have been regarded as unreliable respondents and a number of studies have relied on using proxy measures. Hence data was collected from their parents. This approach is not free from limitations, especially in relation to its accuracy. Children and parents may not share the same views about illness and health [6]. Consequently it has been advocated that children should be asked directly about the impact of illness and health on their daily lives [28]. Another important consideration is the mode of administration of quality of life measures. Self-completed questionnaires are cost effective but may be more suitable for older children. Face to face interviews can be used on younger children. This is more costly but compliance is higher. Hence, there should be a balance between being as comprehensive as possible yet ensuring that a measure is sufficiently succinct so that the instrument can be practically administered [29].

## Conclusion

This study has shown that a brief, direct, interviewer-administered OHRQoL instrument can provide useful data on the oral health-related quality of life of children. Overall, the Child-OIDP showed good reliability and excellent validity. Oral health-related quality of life measurements are aimed at complementing clinical indicators, which has a two-fold advantage. They are useful in moving the focus of provision of health services to patient's perceived needs and quality of life. Thus the provision of dental care on children should address not just their clinical dental need, but give attention to their sociodental needs, taking also into consideration their perceptions in terms of the impact of the oral conditions on their daily life. Also, dental professionals and the public can gain a better understanding of the origins of illnesses. Thus patients would be more likely to engage in health promoting behaviour patterns [30]. This is particularly important in children as their experiences in early life may influence their future attitudes and behaviours.

## Competing interests

The author(s) declare that they have no competing interests.

## Authors' contributions

HY was responsible for the literature review, management and organisation of the study, methodology with assistance from GT, AS, DG, data collection, data input, data analysis and interpretation with assistance from GT and AS. SG was consulted for methodology and analysis and revised the manuscript for its intellectual content. AS contributed to the conception, design and interpretation of the study and critically revised the manuscript and gave final approval for the final version to be published. GT was responsible for substantial contribution to the conception and design of the study, supervised data analysis and interpretation, critically revised the manuscript and gave final approval.
